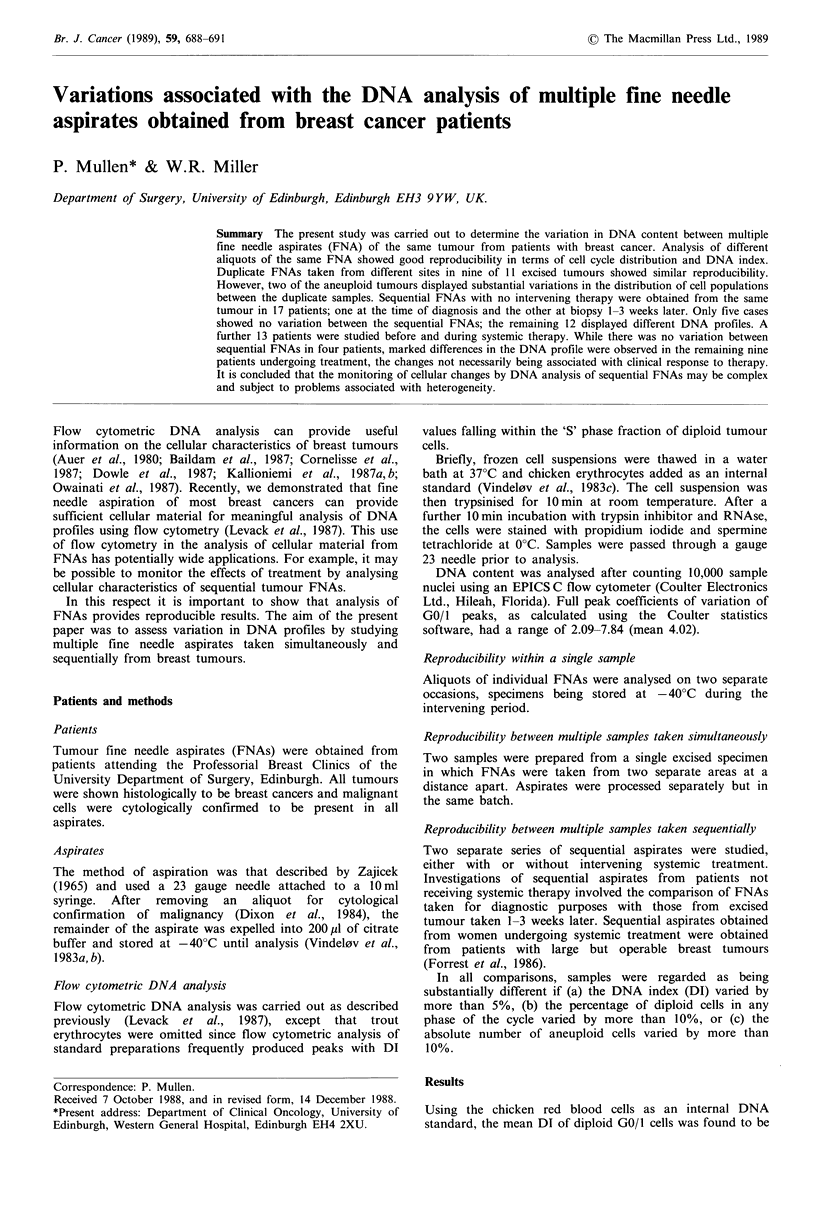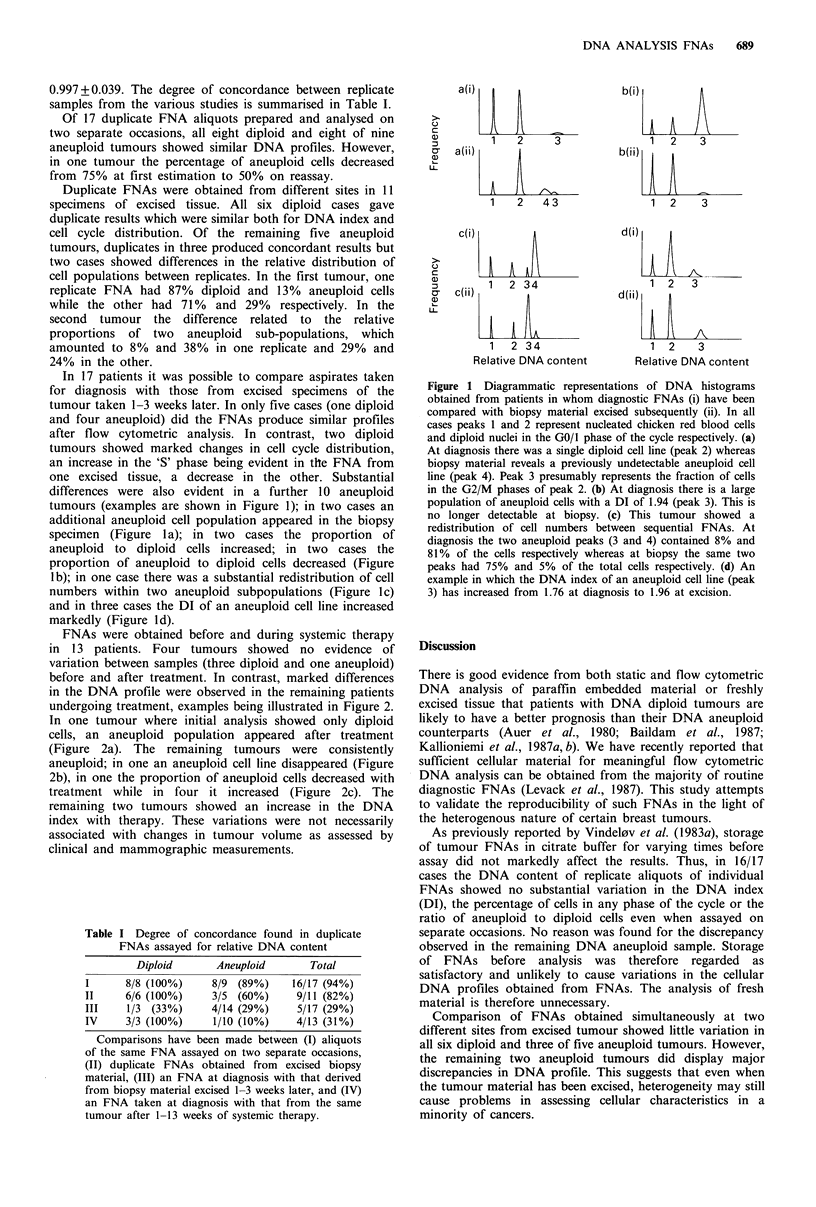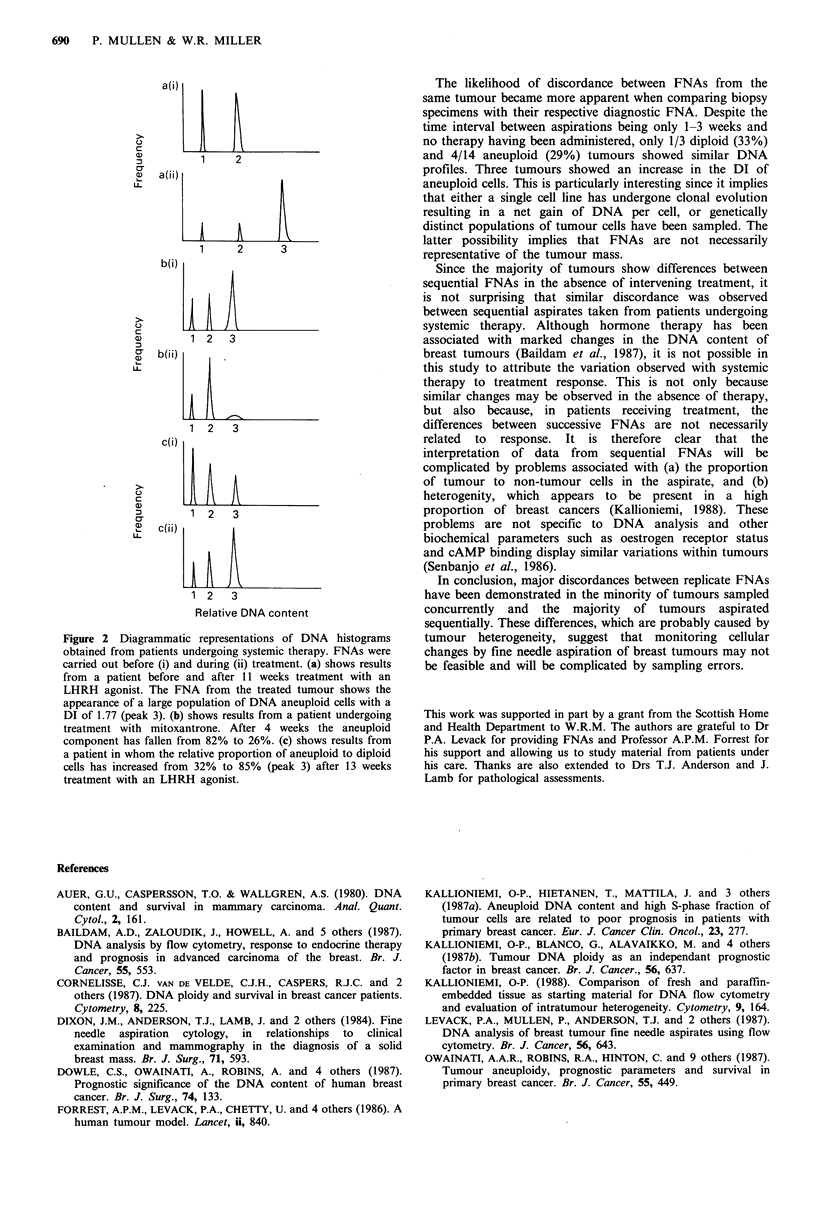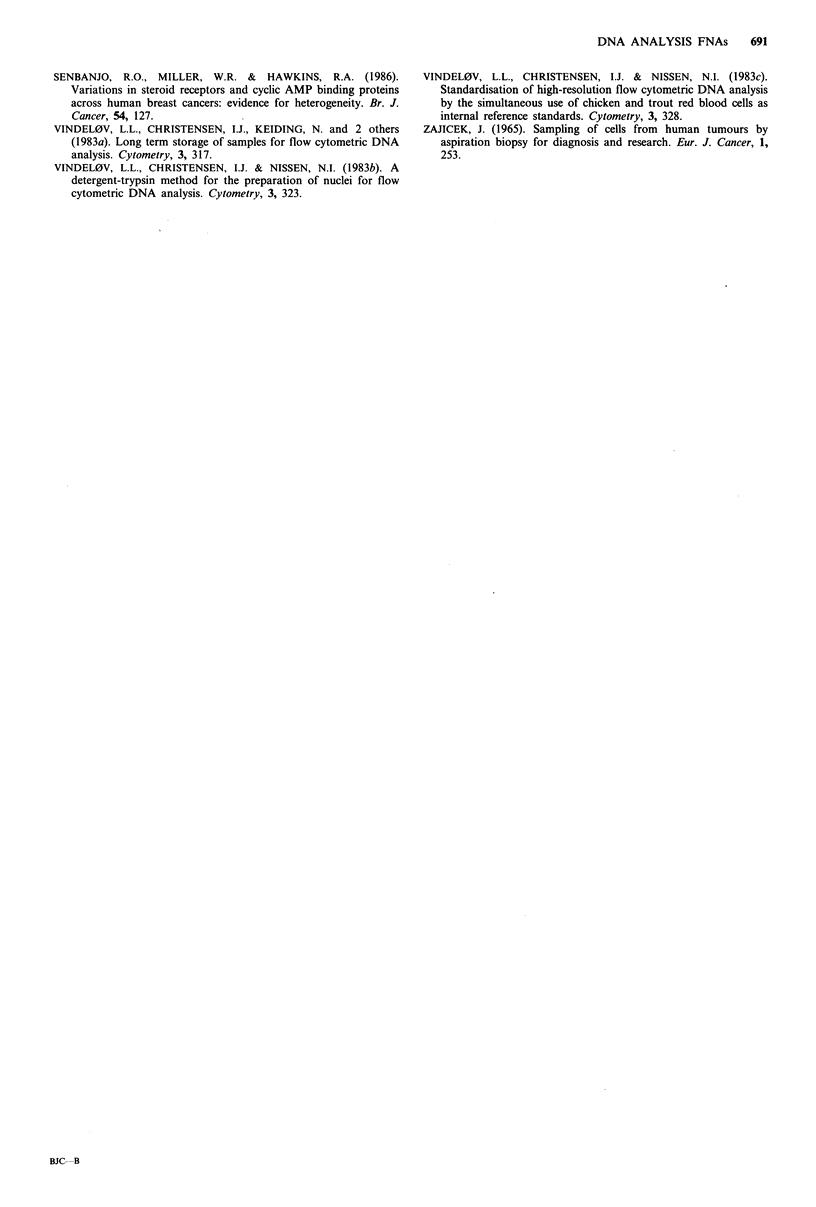# Variations associated with the DNA analysis of multiple fine needle aspirates obtained from breast cancer patients.

**DOI:** 10.1038/bjc.1989.143

**Published:** 1989-05

**Authors:** P. Mullen, W. R. Miller

**Affiliations:** Department of Surgery, University of Edinburgh, UK.

## Abstract

The present study was carried out to determine the variation in DNA content between multiple fine needle aspirates (FNA) of the same tumour from patients with breast cancer. Analysis of different aliquots of the same FNA showed good reproducibility in terms of cell cycle distribution and DNA index. Duplicate FNAs taken from different sites in nine of 11 excised tumours showed similar reproducibility. However, two of the aneuploid tumours displayed substantial variations in the distribution of cell populations between the duplicate samples. Sequential FNAs with no intervening therapy were obtained from the same tumour in 17 patients; one at the time of diagnosis and the other at biopsy 1-3 weeks later. Only five cases showed no variation between the sequential FNAs; the remaining 12 displayed different DNA profiles. A further 13 patients were studied before and during systemic therapy. While there was no variation between sequential FNAs in four patients, marked differences in the DNA profile were observed in the remaining nine patients undergoing treatment, the changes not necessarily being associated with clinical response to therapy. It is concluded that the monitoring of cellular changes by DNA analysis of sequential FNAs may be complex and subject to problems associated with heterogenecity.


Br. J. Cancer (1989), 59, 688–691 © The Macmillan Press Ltd., 1989

### Variations associated with the DNA analysis of multiple fine needle aspirates obtained from breast cancer patients

P. Mullen* & W.R. Miller

*Department of Surgery, University of Edinburgh, Edinburgh EH3 9YW, UK.*

**Summary** The present study was carried out to determine the variation in DNA content between multiple fine needle aspirates (FNA) of the same tumour from patients with breast cancer. Analysis of different aliquots of the same FNA showed good reproducibility in terms of cell cycle distribution and DNA index. Duplicate FNAs taken from different sites in nine of 11 excised tumours showed similar reproducibility. However, two of the aneuploid tumours displayed substantial variations in the distribution of cell populations between the duplicate samples. Sequential FNAs with no intervening therapy were obtained from the same tumour in 17 patients; one at the time of diagnosis and the other at biopsy 1–3 weeks later. Only five cases showed no variation between the sequential FNAs; the remaining 12 displayed different DNA profiles. A further 13 patients were studied before and during systemic therapy. While there was no variation between sequential FNAs in four patients, marked differences in the DNA profile were observed in the remaining nine patients undergoing treatment, the changes not necessarily being associated with clinical response to therapy. It is concluded that the monitoring of cellular changes by DNA analysis of sequential FNAs may be complex and subject to problems associated with heterogeneity.

Flow cytometric DNA analysis can provide useful information on the cellular characteristics of breast tumours (Auer *et al.*, 1980; Baildam *et al.*, 1987; Cornelisse *et al.*, 1987; Dowle *et al.*, 1987; Kallioniemi *et al.*, 1987*a,b*; Owainati *et al.*, 1987). Recently, we demonstrated that fine needle aspiration of most breast cancers can provide sufficient cellular material for meaningful analysis of DNA profiles using flow cytometry (Levack *et al.*, 1987). This use of flow cytometry in the analysis of cellular material from FNAs has potentially wide applications. For example, it may be possible to monitor the effects of treatment by analysing cellular characteristics of sequential tumour FNAs.

In this respect it is important to show that analysis of FNAs provides reproducible results. The aim of the present paper was to assess variation in DNA profiles by studying multiple fine needle aspirates taken simultaneously and sequentially from breast tumours.

### Patients and methods

### *Patients*

Tumour fine needle aspirates (FNAs) were obtained from patients attending the Professorial Breast Clinics of the University Department of Surgery, Edinburgh. All tumours were shown histologically to be breast cancers and malignant cells were cytologically confirmed to be present in all aspirates.

### *Aspirates*

The method of aspiration was that described by Zajicek (1965) and used a 23 gauge needle attached to a 10 ml syringe. After removing an aliquot for cytological confirmation of malignancy (Dixon *et al.*, 1984), the remainder of the aspirate was expelled into 200 $\mu$l of citrate buffer and stored at −40°C until analysis (Vindeløv *et al.*, 1983*a, b*).

### *Flow cytometric DNA analysis*

Flow cytometric DNA analysis was carried out as described previously (Levack *et al.*, 1987), except that trout erythrocytes were omitted since flow cytometric analysis of standard preparations frequently produced peaks with DI values falling within the 'S' phase fraction of diploid tumour cells.

Briefly, frozen cell suspensions were thawed in a water bath at 37°C and chicken erythrocytes added as an internal standard (Vindeløv *et al.*, 1983*c*). The cell suspension was then trypsinised for 10 min at room temperature. After a further 10 min incubation with trypsin inhibitor and RNAse, the cells were stained with propidium iodide and spermine tetrachloride at 0°C. Samples were passed through a gauge 23 needle prior to analysis.

DNA content was analysed after counting 10,000 sample nuclei using an EPICS C flow cytometer (Coulter Electronics Ltd., Hileah, Florida). Full peak coefficients of variation of G0/1 peaks, as calculated using the Coulter statistics software, had a range of 2.09–7.84 (mean 4.02).

### *Reproducibility within a single sample*

Aliquots of individual FNAs were analysed on two separate occasions, specimens being stored at −40°C during the intervening period.

### *Reproducibility between multiple samples taken simultaneously*

Two samples were prepared from a single excised specimen in which FNAs were taken from two separate areas at a distance apart. Aspirates were processed separately but in the same batch.

### *Reproducibility between multiple samples taken sequentially*

Two separate series of sequential aspirates were studied, either with or without intervening systemic treatment. Investigations of sequential aspirates from patients not receiving systemic therapy involved the comparison of FNAs taken for diagnostic purposes with those from excised tumour taken 1–3 weeks later. Sequential aspirates obtained from women undergoing systemic treatment were obtained from patients with large but operable breast tumours (Forrest *et al.*, 1986).

In all comparisons, samples were regarded as being substantially different if (a) the DNA index (DI) varied by more than 5%, (b) the percentage of diploid cells in any phase of the cycle varied by more than 10%, or (c) the absolute number of aneuploid cells varied by more than 10%.

### Results

Using the chicken red blood cells as an internal DNA standard, the mean DI of diploid G0/1 cells was found to be

Correspondence: P. Mullen.
Received 7 October 1988, and in revised form, 14 December 1988.
*Present address: Department of Clinical Oncology, University of Edinburgh, Western General Hospital, Edinburgh EH4 2XU.

$0.997 \pm 0.039$. The degree of concordance between replicate samples from the various studies is summarised in Table I.

Of 17 duplicate FNA aliquots prepared and analysed on two separate occasions, all eight diploid and eight of nine aneuploid tumours showed similar DNA profiles. However, in one tumour the percentage of aneuploid cells decreased from 75% at first estimation to 50% on reassay.

Duplicate FNAs were obtained from different sites in 11 specimens of excised tissue. All six diploid cases gave duplicate results which were similar both for DNA index and cell cycle distribution. Of the remaining five aneuploid tumours, duplicates in three produced concordant results but two cases showed differences in the relative distribution of cell populations between replicates. In the first tumour, one replicate FNA had 87% diploid and 13% aneuploid cells while the other had 71% and 29% respectively. In the second tumour the difference related to the relative proportions of two aneuploid sub-populations, which amounted to 8% and 38% in one replicate and 29% and 24% in the other.

In 17 patients it was possible to compare aspirates taken for diagnosis with those from excised specimens of the tumour taken 1–3 weeks later. In only five cases (one diploid and four aneuploid) did the FNAs produce similar profiles after flow cytometric analysis. In contrast, two diploid tumours showed marked changes in cell cycle distribution, an increase in the 'S' phase being evident in the FNA from one excised tissue, a decrease in the other. Substantial differences were also evident in a further 10 aneuploid tumours (examples are shown in Figure 1); in two cases an additional aneuploid cell population appeared in the biopsy specimen (Figure 1a); in two cases the proportion of aneuploid to diploid cells increased; in two cases the proportion of aneuploid to diploid cells decreased (Figure 1b); in one case there was a substantial redistribution of cell numbers within two aneuploid subpopulations (Figure 1c) and in three cases the DI of an aneuploid cell line increased markedly (Figure 1d).

FNAs were obtained before and during systemic therapy in 13 patients. Four tumours showed no evidence of variation between samples (three diploid and one aneuploid) before and after treatment. In contrast, marked differences in the DNA profile were observed in the remaining patients undergoing treatment, examples being illustrated in Figure 2. In one tumour where initial analysis showed only diploid cells, an aneuploid population appeared after treatment (Figure 2a). The remaining tumours were consistently aneuploid; in one an aneuploid cell line disappeared (Figure 2b), in one the proportion of aneuploid cells decreased with treatment while in four it increased (Figure 2c). The remaining two tumours showed an increase in the DNA index with therapy. These variations were not necessarily associated with changes in tumour volume as assessed by clinical and mammographic measurements.

**Table I** Degree of concordance found in duplicate FNAs assayed for relative DNA content

| | *Diploid* | *Aneuploid* | *Total* |
|---|---|---|---|
| I | 8/8 (100%) | 8/9 (89%) | 16/17 (94%) |
| II | 6/6 (100%) | 3/5 (60%) | 9/11 (82%) |
| III | 1/3 (33%) | 4/14 (29%) | 5/17 (29%) |
| IV | 3/3 (100%) | 1/10 (10%) | 4/13 (31%) |

Comparisons have been made between (I) aliquots of the same FNA assayed on two separate occasions, (II) duplicate FNAs obtained from excised biopsy material, (III) an FNA at diagnosis with that derived from biopsy material excised 1–3 weeks later, and (IV) an FNA taken at diagnosis with that from the same tumour after 1–13 weeks of systemic therapy.

**Figure 1** Diagrammatic representations of DNA histograms obtained from patients in whom diagnostic FNAs (i) have been compared with biopsy material excised subsequently (ii). In all cases peaks 1 and 2 represent nucleated chicken red blood cells and diploid nuclei in the G0/1 phase of the cycle respectively. (**a**) At diagnosis there was a single diploid cell line (peak 2) whereas biopsy material reveals a previously undetectable aneuploid cell line (peak 4). Peak 3 presumably represents the fraction of cells in the G2/M phases of peak 2. (**b**) At diagnosis there is a large population of aneuploid cells with a DI of 1.94 (peak 3). This is no longer detectable at biopsy. (**c**) This tumour showed a redistribution of cell numbers between sequential FNAs. At diagnosis the two aneuploid peaks (3 and 4) contained 8% and 81% of the cells respectively whereas at biopsy the same two peaks had 75% and 5% of the total cells respectively. (**d**) An example in which the DNA index of an aneuploid cell line (peak 3) has increased from 1.76 at diagnosis to 1.96 at excision.

### Discussion

There is good evidence from both static and flow cytometric DNA analysis of paraffin embedded material or freshly excised tissue that patients with DNA diploid tumours are likely to have a better prognosis than their DNA aneuploid counterparts (Auer *et al.*, 1980; Baildam *et al.*, 1987; Kallioniemi *et al.*, 1987*a*, *b*). We have recently reported that sufficient cellular material for meaningful flow cytometric DNA analysis can be obtained from the majority of routine diagnostic FNAs (Levack *et al.*, 1987). This study attempts to validate the reproducibility of such FNAs in the light of the heterogenous nature of certain breast tumours.

As previously reported by Vindeløv *et al.* (1983*a*), storage of tumour FNAs in citrate buffer for varying times before assay did not markedly affect the results. Thus, in 16/17 cases the DNA content of replicate aliquots of individual FNAs showed no substantial variation in the DNA index (DI), the percentage of cells in any phase of the cycle or the ratio of aneuploid to diploid cells even when assayed on separate occasions. No reason was found for the discrepancy observed in the remaining DNA aneuploid sample. Storage of FNAs before analysis was therefore regarded as satisfactory and unlikely to cause variations in the cellular DNA profiles obtained from FNAs. The analysis of fresh material is therefore unnecessary.

Comparison of FNAs obtained simultaneously at two different sites from excised tumour showed little variation in all six diploid and three of five aneuploid tumours. However, the remaining two aneuploid tumours did display major discrepancies in DNA profile. This suggests that even when the tumour material has been excised, heterogeneity may still cause problems in assessing cellular characteristics in a minority of cancers.

**Figure 2** Diagrammatic representations of DNA histograms obtained from patients undergoing systemic therapy. FNAs were carried out before (i) and during (ii) treatment. (**a**) shows results from a patient before and after 11 weeks treatment with an LHRH agonist. The FNA from the treated tumour shows the appearance of a large population of DNA aneuploid cells with a DI of 1.77 (peak 3). (**b**) shows results from a patient undergoing treatment with mitoxantrone. After 4 weeks the aneuploid component has fallen from 82% to 26%. (**c**) shows results from a patient in whom the relative proportion of aneuploid to diploid cells has increased from 32% to 85% (peak 3) after 13 weeks treatment with an LHRH agonist.

The likelihood of discordance between FNAs from the same tumour became more apparent when comparing biopsy specimens with their respective diagnostic FNA. Despite the time interval between aspirations being only 1–3 weeks and no therapy having been administered, only 1/3 diploid (33%) and 4/14 aneuploid (29%) tumours showed similar DNA profiles. Three tumours showed an increase in the DI of aneuploid cells. This is particularly interesting since it implies that either a single cell line has undergone clonal evolution resulting in a net gain of DNA per cell, or genetically distinct populations of tumour cells have been sampled. The latter possibility implies that FNAs are not necessarily representative of the tumour mass.

Since the majority of tumours show differences between sequential FNAs in the absence of intervening treatment, it is not surprising that similar discordance was observed between sequential aspirates taken from patients undergoing systemic therapy. Although hormone therapy has been associated with marked changes in the DNA content of breast tumours (Baildam *et al.*, 1987), it is not possible in this study to attribute the variation observed with systemic therapy to treatment response. This is not only because similar changes may be observed in the absence of therapy, but also because, in patients receiving treatment, the differences between successive FNAs are not necessarily related to response. It is therefore clear that the interpretation of data from sequential FNAs will be complicated by problems associated with (a) the proportion of tumour to non-tumour cells in the aspirate, and (b) heterogenity, which appears to be present in a high proportion of breast cancers (Kallioniemi, 1988). These problems are not specific to DNA analysis and other biochemical parameters such as oestrogen receptor status and cAMP binding display similar variations within tumours (Senbanjo *et al.*, 1986).

In conclusion, major discordances between replicate FNAs have been demonstrated in the minority of tumours sampled concurrently and the majority of tumours aspirated sequentially. These differences, which are probably caused by tumour heterogeneity, suggest that monitoring cellular changes by fine needle aspiration of breast tumours may not be feasible and will be complicated by sampling errors.

This work was supported in part by a grant from the Scottish Home and Health Department to W.R.M. The authors are grateful to Dr P.A. Levack for providing FNAs and Professor A.P.M. Forrest for his support and allowing us to study material from patients under his care. Thanks are also extended to Drs T.J. Anderson and J. Lamb for pathological assessments.

### References

AUER, G.U., CASPERSSON, T.O. & WALLGREN, A.S. (1980). DNA content and survival in mammary carcinoma. *Anal. Quant. Cytol.*, **2**, 161.

BAILDAM, A.D., ZALOUDIK, J., HOWELL, A. and 5 others (1987). DNA analysis by flow cytometry, response to endocrine therapy and prognosis in advanced carcinoma of the breast. *Br. J. Cancer*, **55**, 553.

CORNELISSE, C.J. VAN DE VELDE, C.J.H., CASPERS, R.J.C. and 2 others (1987). DNA ploidy and survival in breast cancer patients. *Cytometry*, **8**, 225.

DIXON, J.M., ANDERSON, T.J., LAMB, J. and 2 others (1984). Fine needle aspiration cytology, in relationships to clinical examination and mammography in the diagnosis of a solid breast mass. *Br. J. Surg.*, **71**, 593.

DOWLE, C.S., OWAINATI, A., ROBINS, A. and 4 others (1987). Prognostic significance of the DNA content of human breast cancer. *Br. J. Surg.*, **74**, 133.

FORREST, A.P.M., LEVACK, P.A., CHETTY, U. and 4 others (1986). A human tumour model. *Lancet*, **ii**, 840.

KALLIONIEMI, O-P., HIETANEN, T., MATTILA, J. and 3 others (1987*a*). Aneuploid DNA content and high S-phase fraction of tumour cells are related to poor prognosis in patients with primary breast cancer. *Eur. J. Cancer Clin. Oncol.*, **23**, 277.

KALLIONIEMI, O-P., BLANCO, G., ALAVAIKKO, M. and 4 others (1987*b*). Tumour DNA ploidy as an independant prognostic factor in breast cancer. *Br. J. Cancer.*, **56**, 637.

KALLIONIEMI, O-P. (1988). Comparison of fresh and paraffin-embedded tissue as starting material for DNA flow cytometry and evaluation of intratumour heterogeneity. *Cytometry*, **9**, 164.

LEVACK, P.A., MULLEN, P., ANDERSON, T.J. and 2 others (1987). DNA analysis of breast tumour fine needle aspirates using flow cytometry. *Br. J. Cancer*, **56**, 643.

OWAINATI, A.A.R., ROBINS, R.A., HINTON, C. and 9 others (1987). Tumour aneuploidy, prognostic parameters and survival in primary breast cancer. *Br. J. Cancer*, **55**, 449.

SENBANJO, R.O., MILLER, W.R. & HAWKINS, R.A. (1986). Variations in steroid receptors and cyclic AMP binding proteins across human breast cancers: evidence for heterogeneity. *Br. J. Cancer*, **54**, 127.

VINDELØV, L.L., CHRISTENSEN, I.J., KEIDING, N. and 2 others (1983*a*). Long term storage of samples for flow cytometric DNA analysis. *Cytometry*, **3**, 317.

VINDELØV, L.L., CHRISTENSEN, I.J. & NISSEN, N.I. (1983*b*). A detergent-trypsin method for the preparation of nuclei for flow cytometric DNA analysis. *Cytometry*, **3**, 323.

VINDELØV, L.L., CHRISTENSEN, I.J. & NISSEN, N.I. (1983*c*). Standardisation of high-resolution flow cytometric DNA analysis by the simultaneous use of chicken and trout red blood cells as internal reference standards. *Cytometry*, **3**, 328.

ZAJICEK, J. (1965). Sampling of cells from human tumours by aspiration biopsy for diagnosis and research. *Eur. J. Cancer*, **1**, 253.